# Inpatient Cognitive Behavior Therapy for Adolescents with Anorexia Nervosa: Immediate and Longer-Term Effects

**DOI:** 10.3389/fpsyt.2014.00014

**Published:** 2014-02-12

**Authors:** Riccardo Dalle Grave, Simona Calugi, Marwan El Ghoch, Maddalena Conti, Christopher G. Fairburn

**Affiliations:** ^1^Department of Eating and Weight Disorders, Villa Garda Hospital, Garda, Italy; ^2^Department of Psychiatry, Warneford Hospital, Oxford University, Oxford, UK

**Keywords:** anorexia nervosa, eating disorders, adolescents, cognitive behavior therapy, family therapy, treatment, hospitalization, relapse

## Abstract

**Introduction:** Inpatient treatment for anorexia nervosa is often successful in restoring body weight, but a high percentage of patients relapse following discharge. The aim of the present study was to establish the immediate and longer-term effects of a novel inpatient program for adolescents that was designed to produce enduring change.

**Method:** Twenty-seven consecutive patients with severe anorexia nervosa were admitted to a 20-week inpatient treatment program based on the enhanced cognitive behavior therapy (CBT-E). The patients were assessed before and after hospitalization, and 6 and 12 months later.

**Results:** Twenty-six patients (96%) completed the program. In these patients, there was a substantial improvement in weight, eating disorder features, and general psychopathology that was well maintained at 12-month follow-up.

**Conclusion:** These findings suggest that inpatient CBT-E is a promising approach to the treatment of adolescents with severe anorexia nervosa.

## Introduction

Adolescents with anorexia nervosa are more vulnerable to the effects of malnutrition and weight loss than adults as their organs are not yet fully developed. Indeed, if the weight restoration is delayed or incomplete, the physical damage, in particular osteoporosis, growth arrest, and absent puberty, may be irreversible (1, 2). Anorexia nervosa in adolescents is also associated with developmental psychological regression and parental dependence (3). In the most severe cases, patients become socially isolated, disaffected with most of their former interests, and persistence of the disorder may even damage the development of their identity (4) and increase the risk of death (5).

These facts justify a low threshold for active intervention in young adolescent patients with anorexia nervosa, and if weight regain does not occur with outpatient treatment within a reasonable time frame, hospitalization needs to be seriously considered. This is not a rare outcome since family based treatment (FBT), the leading empirically supported outpatient intervention (6), produces a full treatment response in fewer than half the patients (7).

Few data are available on the effect of inpatient treatment for adolescents with severe anorexia nervosa (8). The available studies indicate that it is associated with many short-term benefits, such as the restoration of healthy body weight, the improvement of eating disorder, and general psychopathology (9, 10). However, the longer-term effects are less clear-cut. A study of 60 consecutive adolescent patients found that 32% met criteria for a full or partial eating disorder at an average length of post-hospitalization follow-up of 58 months (11). Another study found that those who had received inpatient treatment had a significantly worse outcome than those never admitted to hospital (12). These findings are consistent with those from studies of adults (13–16) in indicating that the main limitation of inpatient treatment of severe anorexia nervosa is the high rate of relapse following discharge.

Post-hospitalization methods for reducing the rate of relapse have been tested in adults with anorexia nervosa. A report suggested that fluoxetine might have this effect (17), but a subsequent controlled trial did not confirm this (15). Other reports suggest that cognitive behavior therapy (CBT) might be beneficial (18, 19), but this remains to be substantiated.

In this study, we modified certain aspects of a conventional inpatient treatment program with the aim of improving outcome and reducing patients’ propensity to relapse on discharge. We replaced the traditional eclectic approach to inpatient treatment with a treatment exclusively based on “enhanced” CBT (CBT-E) for eating disorders (20). The rational for this decision was as follows. First, CBT-E is designed to produce enduring change through the modification of the key mechanisms thought to maintain eating disorder psychopathology (i.e., undereating and underweight, events and moods influencing eating, and overevaluation of shape and weight) using a flexible series of sequential cognitive behavioral procedures and strategies, integrated with progressive patient education, and the development of personalized relapse prevention skills (20). Second, outpatient CBT-E has been shown to produce a sustained change in patients with bulimia nervosa or eating disorder not otherwise specified in three independent studies (combined *n* = 315) (21–23) and in two cohorts of adults with anorexia nervosa (total *n* = 99) (24) as well as a cohort of adolescents (*n* = 47) (25).

This paper reports the immediate and longer-term effects of this new inpatient CBT-E program. The specific aim was to address three key clinical questions. First, among adolescents with anorexia nervosa, what proportion is able to complete inpatient CBT-E? Second, among the patients who do complete inpatient CBT-E, what is their outcome? Third, how well are the changes maintained following inpatient CBT-E?

## Materials and Methods

### Design

A cohort of adolescent patients with severe anorexia nervosa was recruited. Eligible patients were offered 20 weeks of treatment, the first 13 weeks being on an inpatient basis and the remaining 7 being as a day patient. Patients were assessed before treatment, after the end of treatment, and 6 and 12 months later. The ethics committee of the Local Health Unit 22-Bussolengo approved the study (Study Protocol No. 86496 USL22, approved 20/12/05), and the patient’s parents or legal guardians gave written informed consent to participation and to the anonymous use of personal data after having received a full description of the study.

### Recruitment

The sample was recruited from consecutive referrals to the eating disorder inpatient unit of Villa Garda Hospital (Northern Italy). The source of referrals was heterogeneous and included family doctors, and secondary care health professionals (i.e., eating disorder specialists, outpatient eating disorder units of the National Italian Health System, general psychiatrists, and acute internal medicine units). The patients had to be aged between 13 and 17 years and to fulfill the DSM-IV diagnostic criteria for anorexia nervosa (26), bar the amenorrhea criterion, as judged both by the referring clinician and by an eating disorder specialist (Riccardo Dalle Grave), and to require inpatient treatment either as a result of failure of outpatient treatment or because the eating disorder could not be managed safely on an outpatient basis. Twenty-seven out of 32 (84.3%) eligible patients accepted inpatient treatment, and were added to the unit’s waiting list of up to 8 weeks. Of the remaining five patients, one was excluded because of a co-occurring acute psychotic state and another because of significant substance abuse, while three (9.4%) declined to participate. During the period on the waiting list the patients were managed by the referring agency.

### The unit

The unit specializes in the treatment of patients with eating disorder and includes designated spaces for adolescents. Young patients sleep in separate rooms from adults, and have the access at a study room with computers equipped with communications software to allow them to maintain contact with their classmates and significant others. In addition, those who are students received tutoring from a teacher, either directly or via Internet. The unit is “open” (i.e., patients are free to come and go during the day) provided they are judged to be in a stable medical condition. Similarly, patients are free to receive visits from significant others so long as they occur outside treatment sessions. In the day patient phase of treatment families can rent a local apartment if they live too far away from the hospital for the patient to commute.

### Intervention

The treatment is closely based on the CBT-E for eating disorders (20). It has been adapted to make it suitable both for an inpatient setting and for adolescents. The treatment can be considered as a form of intensive psychotherapy. In style, it is not an institutional and medical treatment. The entire inpatient treatment experience consists of CBT-E for 24 h a day, 7 days a week. In other words, it is CBT-E “immersion,” with the unit providing a consistent and coherent therapeutic approach. The treatment is concerned with the patients’ entire functioning (psychological, physical, and social), not just their eating and weight, and it is designed to enhance their control over their eating and life.

Admission to the program is voluntary, both for adults and adolescents. Referred patients are helped to participate in the decision to be admitted in three pre-treatment preparatory sessions conducted along similar lines to the equivalent sessions in outpatient CBT-E (20). The expectation is that the patient will play an active role in treatment.

The program is designed to provide single coherent CBT-E oriented approach to the treatment of the patient’s eating disorder (27–29). Its precise content is dictated by the patient’s “formulation” (case conceptualization), derived at the outset and updated as treatment progresses. The treatment retains all the main therapeutic strategies and procedures of CBT-E and these are mostly delivered in individual sessions from a trained clinical psychologist. These hour-long sessions take place twice a week during the first 4 weeks and once a week thereafter. They focus on the following topics: (i) helping patients adjust to and accept the rapid changes in shape and weight and address the overevaluation of shape and weight; (ii) helping them deal with events and moods affected their eating; (iii) if indicated, addressing clinical perfectionism, core low self-esteem, or interpersonal difficulties; (iv) preparing a post-discharge treatment plan in order to achieve a smooth transition from inpatient to outpatient treatment. In addition, there is assistance with eating in the early stages of treatment until patients achieve a body mass index (BMI) centile corresponding at a BMI ≥ 18.5 (28). There are also CBT-E-based group sessions (four times a week) focused on addressing dietary restraint, body image disturbance, and mood intolerance. Lastly, there are group physical exercise sessions twice weekly to help restore muscle mass and improve fitness. Further details of the program have been provided elsewhere (27–29).

With adolescent patients the inpatient program includes two additional elements (29). First, the parents participate in a CBT-E-based family module that included six sessions with the psychologist delivering the individual CBT-E and two sessions with a CBT-E trained dietitian. There is also a parents-only session in the first week of treatment dedicated to the assessment of the family environment and to educating parents about their child’s eating disorder and the processes that are likely to be maintaining it. The aim is to identify potential family factors that might hinder the patient’s efforts to change. The remaining five sessions are designed to improve communication between family members, to develop functional strategies for managing crises, and to create positive home environment that is likely to support the patient’s efforts to change. The sessions with the dietitian are designed to help plan meals at home. The second additional element are weekly group sessions addressing general adolescent issues including identity, autonomy, the development of social skills, and coping with puberty.

Outpatient-based CBT-E includes many strategies and procedures designed to ensure that the changes made during the treatment are well maintained (20). The inpatient program includes three additional features designed to reduce the high rate of relapse that typically follows discharge from hospital (28). First, the inpatient unit is “open.” In this way patients continue to be exposed to the types of environmental stimuli that tend to provoke the return of eating disorder psychopathology. Second, during the weeks prior to discharge, effort is made on a case-by-case basis to identify the likely triggers of setbacks. These are then addressed in the individual CBT-E sessions. Third, significant others are helped to create a positive stress-free home environment in readiness for the patient’s return.

### Assessment

#### Body weight and body mass index

Weight was measured using a beam balance scale and height was measured using a wall-mounted stadiometer. BMI centiles were calculated using the Center for Disease Control and Prevention growth charts (www.cdc.gov/growthcharts).

#### Eating disorder features

They were assessed using the Italian version of 12th edition of the Eating Disorder Examination (EDE) interview adapted for children (12.OD/C4) (30, 31). The EDE was administered by assessors who were trained and supervised by Riccardo Dalle Grave, an expert on the instrument.

#### General psychiatric features

These were measured using the Brief Symptom Inventory (BSI) (32, 33), a short version of the Symptom Checklist-90 (34).

### Statistical analysis

Data are presented as *N* (%) for categorical data and as means (with standard deviation, SD) or medians (with range) for continuous data. Repeated measures analysis of variance on continuous variables and Bonferroni *post hoc* tests were performed to verify significant changes over time on clinical variables. All statistical analyses were carried out by SPSS version 20.0 (SPSS Inc., Chicago).

## Results

### The sample

The mean age of the 27 patients was 16.0 years (SD 1.1, range 13–17 years). All the patients were Caucasian and single, and just one was male. The mean duration of the eating disorder was 2.0 years (range 0–7, median 1.0 years). The patients were substantially underweight, with 16 (59.6%) having a BMI centile of <1. The mean (SD) BMI centile (taking BMI centile as 0.5 for those with a value <1) was 2.7 (SD 4.3, range 0.5–14.4, median 0.5). All the patients received at least one previous outpatient treatment, and five patients had previous hospitalization in internal medical departments.

### Intent-to-treat findings at the end of treatment and at 6 and 12-month follow-up

Intent-to-treat data moving the last available data point forward, are reported in Table 1. There was a marked increase in weight. By the end of treatment the mean BMI centile had increased from 2.7 (SD 4.3) to 34.2 (SD 15.7). At 6-month follow-up the mean BMI centile was 27.3 (SD 20.8) and at 12-month follow-up it was 29.9 (SD 20.1) when 81.5% (22/27) had a BMI centile corresponding at a BMI ≥ 18.5.

**Table 1:** **Characteristics of the patients before treatment, after treatment, and at 6- and 12-month follow-up (intent-to-treat data set, *n* = 27)**.

	Before treatment	After treatment	6-month follow-up	12-month follow-up
Weight
Body weight (kg)	38.5 (6.1)	49.7 (5.6)^a^	45.7 (7.0)^a^^,^^b^	48.1 (7.3)^a^
Body mass index centile	2.7 (4.2)	34.2 (15.7)^a^	27.3 (20.8)^a^^,^^b^	29.9 (20.1)^a^
Eating disorder psychopathology
Overall severity (global EDE)	3.7 (1.3)	2.1 (1.2)^a^	2.1 (1.7)^a^	1.7 (1.3)^a^
Global EDE <1 SD above the community mean^d^, *n* (%)	2 (7.4)	10 (37.0)	14 (51.9)	14 (51.9)
Dietary restraint (EDE subscale)	4.1 (1.2)	1.1 (1.1)^a^	1.8 (1.9)^a^	1.2 (1.5)^a^
Eating concern (EDE subscale)	3.3 (1.4)	1.5 (1.4)^a^	1.7 (1.6)^a^	1.4 (1.2)^a^
Shape concern (EDE subscale)	3.8 (1.8)	3.2 (1.4)	2.9 (1.8)	2.5 (1.7)^a^
Weight concern (EDE subscale)	3.5 (1.9)	2.4 (1.4)^a^	2.1 (1.8)^a^	1.9 (1.5)^a^
Eating disorder behavior (EDE)
Binge eating, *n* (%) present	8 (29.6)	2 (7.4)	5 (18.5)	4 (14.8)
If present, episodes/28 days, median (range)	17 (2–148)	5 (1–9)	15 (1–30)	7 (1–15)
Self-induced vomiting, *n* (%) present	10 (37.0)	4 (14.8)	7 (25.9)	7 (25.9)
If present, episodes/28 days, median (range)	25 (1–196)	1.5 (1–9)	20 (1–40)	3 (1–28)
Laxative misuse, *n* (%) present	3 (11.1%)	0	1 (3.7%)	0
If present, episodes/28 days, median (range)	1 (1–20)	–	1	–
General psychiatric features, GSI	1.8 (0.8)	1.0 (0.7)^a^	1.2 (0.9)^a^	0.8 (0.7)^a^^,^^c^

*EDE, eating disorder examination (version 15.0 D); GSI, global severity index*.

*^a^*p* < 0.05 vs. baseline*.

*^b^*p* < 0.05 vs. end of therapy*.

*^c^*p* < 0.05 vs. 6-month follow-up*.

*^d^Global EDE <1 SD above community EDE mean for young adult women (i.e., below 1.74)*.

*Data are shown as mean (SD) unless otherwise stated*.

There was also marked decrease in the level of eating disorder and general psychopathology from baseline to discharge (EDE Global score: *t* = 5.36, *p* < 0.001; BSI-GSI: *t* = 8.29, *p* < 0.001) that was maintained both at 6- and 12-month follow-up (EDE Global score from discharge to 6- and 12-month follow-up: *t* = −0.92, *p* = 0.368; *t* = 1.11, *p* = 0.277, respectively; BSI-GSI from discharge to 6- and 12-month follow-up: *t* = −0.22, *p* = 0.831; *t* = 1.19, *p* = 0.246, respectively).

### Question 1 – what proportion of patients was able to complete this inpatient CBT-E program?

One patient (3.7%, 1/27) did not complete the program. This was because of sustained lack of progress.

### Question 2 – what was the outcome among those who completed CBT-E?

There was a substantial increase in weight among the 26 treatment completers (see Table 2). The mean weight gain from admission to discharge was 11.7 kg (SD 3.6; 95% CI 10.2–13.2; *p* < 0.001), equivalent to a BMI centile increase of 32.7 (SD 12.8; 95% CI 27.5–37.9; *p* < 0.001). Twenty-five patients (96.2%) achieved a BMI centile corresponding at a BMI ≥ 18.5. Eating disorder psychopathology and general psychiatric features also improved substantially with the mean global EDE score decreasing by 1.7 (SD 1.0; 95% CI 1.3–2.1; *p* < 0.001) and the mean BSI-GSI decreasing by 0.9 (SD 0.8; 95% CI 0.6–1.2; *p* < 0.001). Almost 40% of patients (38.5%, 10/26) had minimal residual eating disorder psychopathology, defined as having a global EDE score below 1 SD above the community mean (i.e., <1.74) (35).

**Table 2:** **Characteristics of the patients before treatment, after treatment, and at 6- and 12-month follow-up among those who completed treatment**.

	Before treatment (*n* = 26)	After treatment (*n* = 26)	6-month follow-up (*n* = 22)	12-month follow-up (*n* = 23)
Weight
Body weight (kg)	38.6 (6.1)	50.4 (4.7)^a^	46.4 (6.6)^a^	48.4 (6.8)^a^
Body mass index centile	2.8 (4.3)	35.5 (14.5)^a^	25.4 (20.6)^a^^,^^b^	29.5 (19.6)^a^
Eating disorder psychopathology
Overall severity (global EDE)	3.7 (1.3)	2.0 (1.1)^a^	2.1 (1.7)^a^	1.5 (1.3)^a^
Global EDE <1 SD above the community mean^d^, *n* (%)	2 (7.7)	10 (38.5)^a^	13 (59.1)^a^	9 (60.9)^a^
Dietary restraint (EDE subscale)	4.1 (1.2)	1.0 (1.0)^a^	1.8 (2.0)^a^	1.0 (1.3)^a^
Eating concern (EDE subscale)	3.3 (1.4)	1.5 (1.4)^a^	1.8 (1.7)^a^	1.3 (1.2)^a^
Shape concern (EDE subscale)	3.8 (1.8)	3.2 (1.4)	2.8 (1.8)	2.1 (1.6)^a^
Weight concern (EDE subscale)	3.5 (1.9)	2.3 (1.4)^a^	1.9 (1.8)^a^	1.6 (1.4)^a^
Eating disorder behavior (EDE)
Binge eating, *n* (%) present	8 (30.8)	2 (7.7)	4 (18.2)	3 (13.0)
If present, episodes/28 days, median (range)	17 (2–148)	8 (1–15)	22.5 (1–30)	1 (1–15)
Self-induced vomiting, *n* (%) present	10 (38.5)	4 (15.4)	6 (27.3)	6 (26.1)
If present, episodes/28 days, median (range)	25 (1–196)	10.5 (0–30)	25 (2–40)	4.5 (1–28)
Laxative misuse, *n* (%) present	3 (11.5%)	0	1 (4.5%)	0
If present, episodes/28 days, median (range)	1 (1–20)	–	1	–
General psychiatric features, GSI	1.9 (0.8)	1.0 (0.7)^a^	1.3 (1.0)^a^	0.8 (0.7)^a^^,^^c^

*EDE, eating disorder examination (version 15.0 D); GSI, global severity index*.

*^a^*p* < 0.05 vs. baseline*.

*^b^*p* < 0.05 vs. end of therapy*.

*^c^*p* < 0.05 vs. 6-month follow-up*.

*^d^Global EDE <1 SD above community EDE mean for young adult women (i.e., below 1.74)*.

*Data are shown as mean (SD) unless otherwise stated*.

### Question 3 – were the changes sustained following CBT-E?

There was high compliance with follow-up with 81.5% (22/27) and 85.2% (23/27) of the treatment completers being reassessed at 6- and 12-month follow-up points respectively. Twenty-two (84.6%) patients received post-discharge outpatient treatment. This varied in nature and intensity and was delivered by therapists living close to the patients’ place of residence.

**Figure 1:**
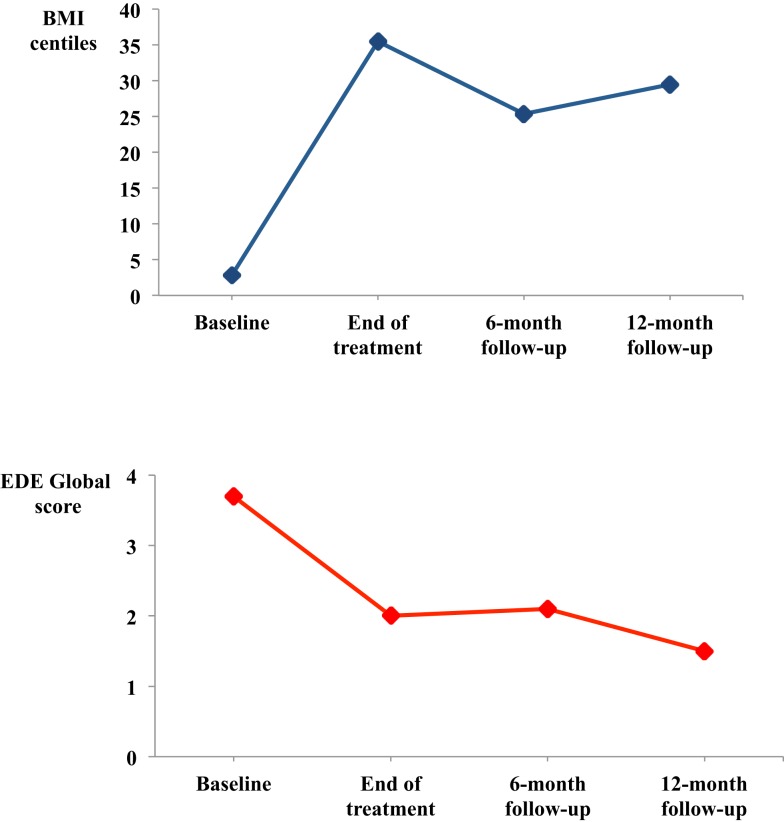
**Mean body mass index (BMI) centile and eating disorder psychopathology (global EDE) over treatment and 12-month of follow-up among those patients who completed inpatient CBT-E (*n* = 26)**.

Overall, the changes made in hospital were well maintained (see Table 2 and Figure 1), and the BMI centile was not significantly different from discharge at 12-month follow-up, when 82.6% (19/23) had a BMI centile corresponding at a BMI ≥ 18.5.

## Discussion

The aim of the present study was to assess the immediate and longer-term effects of a new form of inpatient treatment for adolescents with anorexia nervosa. To this end, a cohort of adolescent patients with severe anorexia nervosa was admitted to a CBT-E-based inpatient program and then reassessed 6 and 12 months later.

The study had three main findings. The first was that the treatment was well accepted by the patients. Almost 85% of eligible patients accepted the treatment and 96% completed the program despite its goal of complete weight restoration.

The second finding was that patients responded well to the treatment. At the end of treatment 96.2% of completers had a BMI centile corresponding at a BMI ≥ 18.5, and the mean BMI centile increased from 2.7 to 38.5. The increase of BMI was accompanied by a marked decrease in eating disorder and general psychopathology.

The third finding was that patients maintained the marked improvements in weight, eating disorder psychopathology, and general psychiatric features achieved during treatment. At 12-month of follow-up 82.6% had a BMI centile corresponding at a BMI ≥ 18.5, a BMI centile not significantly different from discharge. The improvements in eating disorder and general psychopathology were well maintained following the discharge. These findings suggest that inpatient CBT-E for adolescents is not associated with the high rate relapse, that follows traditional inpatient treatment in which more of 70% of adult patients had a BMI lower that 18.5 at 12-month follow-up (36), and only 14% of adolescent patients had a good outcome at 2–7 years of follow-up (12). We speculate that the better outcome observed with our inpatient treatment, although we do not yet know the active elements of the treatment, might be the effect of three main factors. First, the use of CBT-E procedures addressing the key maintaining mechanisms of eating disorder psychopathology, including also the development of personalized relapse prevention skills (20). Second, the involvement of parents in the treatment to create an optimal family environment that is likely to support the patient’s efforts to change. Third, the open nature of the unit that expose the patients to some potential environment triggers of relapse.

The study had some strengths. First, the cohort was small but representative as it was recruited from an inpatient unit of the National Italian Health System. Second, the sample was a severely affected one. About 60% had a BMI centile of <1 and the mean BMI centile was 2.7. Third, CBT-E was the only psychological treatment delivered to the patients during their inpatient stay. Fourth, the patients were followed up for 12-month, the period during which relapse is particularly likely to occur.

The study had also certain limitations. First, the follow-up was not closed. Most patients (*N* = 22) received outpatient treatment after discharge, a fact that complicates the interpretation of the results. However, this limitation is shared by all studies of inpatient treatment yet, despite the subsequent outpatient treatment sessions, relapse is reported to be common (15, 17). Second, the study did not include a comparison condition. This was for logistical reasons it is extremely difficult comparing different inpatient programs.

Future research should evaluate the effectiveness of more economic forms of inpatient CBT-E for adolescents with anorexia nervosa not responding to standard outpatient CBT-E. One promising approach that has yet to be formally evaluated is to address with a shorter inpatient CBT-E only the main key mechanisms responsible for the lack of progress with outpatient CBT-E, and then, once these obstacles have been overcome, to continue the treatment with standard outpatient CBT-E. Common outpatient obstacles are associated with the inability to address dietary restriction and weight regain or reducing the frequency of some eating disorder behaviors (e.g., bulimic episodes, self-induced vomiting, excessive, and compulsive exercising). Finally, there is the need of a direct comparison study with “treatment as usual” in order to evaluate the true efficacy of the inpatient CBT-E.

In conclusion, the findings of this study suggest that inpatient CBT-E is a promising treatment for adolescents with severe anorexia nervosa. The treatment was well accepted and produced a marked improvement in weight and psychopathology that was maintained at 12-month follow-up.

## Author Contributions

All authors have authorship and have made substantial contributions and final approval of the conceptions, drafting, and final version. The study has been designed by Riccardo Dalle Grave and Christopher G. Fairburn. Data assessment was carried by Maddalena Conti and Marwan El Ghoch. The statistical analysis has been performed by Simona Calugi. Riccardo Dalle Grave, Christopher G. Fairburn, and Simona Calugi participated in writing the manuscript.

## Conflict of Interest Statement

The authors declare that the research was conducted in the absence of any commercial or financial relationships that could be construed as a potential conflict of interest.
